# Series of Microporous Redox‐Active Pillared Metal–Organic Frameworks Based On Alloxazine Ligands

**DOI:** 10.1002/open.202500461

**Published:** 2025-09-09

**Authors:** Jaison Casas, Alexios I. Vicatos, Leonard J. Barbour, Nathalie Kyritsakas, Abdelaziz Jouaiti, Sylvie Ferlay

**Affiliations:** ^1^ CMC UMR 7140 CNRS Université de Strasbourg Strasbourg F‐67000 France; ^2^ Department of Chemistry and Polymer Science Stellenbosch University Matieland 7602 South Africa; ^3^ Service de radiocristallographie de la Fédération de Chimie Le Bel – UAR 2042 Université de Strasbourg and CNRS Strasbourg F‐67000 France

**Keywords:** alloxazine, gas adsorption, metal–organic frameworks, pillars, redox‐active

## Abstract

Two series of robust pillared metal–organic frameworks (MOFs) are obtained under solvothermal conditions by combining a metal salt with either **H**_**2**_ **bpdc**, biphenyl‐4,4′‐dicarboxylic acid, or **H**_**2**_ **pda**, 1,4‐phenylenediacrylic acid, forming 2D layers, which are pillared by **L**, an alloxazine derivative of 1,4‐di(pyridin‐4‐yl)benzene using a one‐pot three‐component strategy. Crystallographic studies reveal the formation of two isomorphous series of compounds, namely **1‐M** (from **H**_**2**_ **bpdc** with M = Co, Ni, Cu, and Zn) and **2‐M** (from **H**_**2**_ **pda** with M = Co or Cu). The multifunctional compounds have high decomposition temperatures, and their sorption properties were measured, revealing relatively low surface areas. Furthermore, **1‐Zn** displays a moderate uptake of CO_2_ and C_2_H_4_ at high pressures**.** In addition, for **1‐M** (M = Co, Cu or Zn), solid‐state electrochemistry reveals redox behavior for the MOF, centered on the ligand. This study provides evidence for the first account of a one‐pot formation of redox‐active pillared MOFs, which exhibit gas sorption abilities before the reduction.

## Graphical abstract

Two series of robust pillared metal–organic frameworks using a redoactive ligand based on alloxazine and transition metals have been evidenced by XRD. The compounds have high decomposition temperatures, and their sorption properties are measured, revealing relatively low surface areas and a moderate uptake of CO_2_ and C_2_H_4_ at high pressures**.**

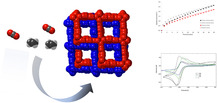

## 1 Introduction

**Figure 1:**
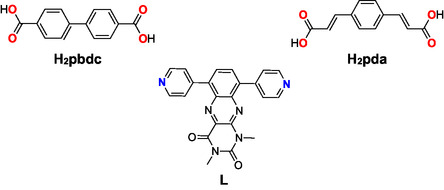
Ligands used for the formation of 2D layers (**H**_**2**_ **pbdc** and **H**_**2**_ **pda**) pillared by **L**, an alloxazine derivative, in order to obtain 3D MOFs.

Metal–organic frameworks (MOFs)^[^ ^1^, ^2^, ^3^ ^]^ have been widely studied and developed, because this class of materials displays structures with permanent porosity, high specific surface areas, and tunable topologies. They offer potential for applications in many fields,^[^ ^4^ ^]^ such as gas storage^[^ ^5^, ^6^, ^7^, ^8^ ^]^ and separation,^[^ ^8^ ^,^ ^9^ ^]^ catalysis,^[^ ^10^ ^,^ ^11^ ^]^ smart materials,^[^ ^12^ ^]^ and drug delivery.^[^ ^13^ ^,^ ^14^ ^]^ More specifically, redox‐active MOFs^[^ ^15^, ^16^, ^17^ ^]^ have attracted particular interest, because of their applicability in gas separation and purification, (electrochemical) sensing, photochromism, (photo)catalysis, electronic conductivity and magnetism, and also energy storage devices (rechargeable batteries and supercapacitors).^[^ ^18^ ^]^ Different strategies can be employed to synthesize redox‐active MOFs, including the exploration of various accessible oxidation states linked to the metal^[^ ^16^ ^]^ or the use of redox‐active ligands and organic redox‐active guests, or combining them. Among the potential redox‐active species that are used either as ligands or as guests for the formation of redox‐active MOFs, alloxazines, isomers of *iso*alloxazine related to flavins,^[^ ^19^ ^]^ belong to an important class of biomolecules exhibiting three different redox and protonation states, including an accessible intermediate radical state.^[^ ^20^ ^]^ Even though alloxazine derivatives are well‐known ligands for transition metals,^[^ ^21^ ^,^ ^22^ ^]^ we have recently reported a groundbreaking redox‐active divergent ligand based on alloxazine,^[^ ^23^ ^]^ and to the best of our knowledge, this includes the creation of the first alloxazine‐based coordination polymer that exhibits redox activity^[^ ^23^ ^]^: a coordination polymer involving an alloxazine based ligand (**L**, **Figure** **1**) was obtained, but unfortunately, the compound is not stable when exposed to the air.

Redox‐active MOFs (RAMOFs) require stability in air and significant thermal resilience to be considered for practical for use or viable as alternatives to traditional inorganic zeolites in commercial applications. To increase the stability of these MOFs,^[^ ^24^ ^]^ several approaches have been developed, one of which is based on the development of “pillared MOFs,”^[^ ^25^, ^26^, ^27^, ^28^ ^]^ (a subclass of Hofmann clathrates^[^ ^29^ ^]^). The “pillared MOFs” strategy is an effective and straightforward approach for the formation of 3D porous frameworks that can be particularly flexible and robust. These materials have shown interesting gas sorption behavior, frequently with gate‐opening effects.^[^ ^30^ ^]^ In addition, it has been recently reported that this gate‐opening phenomenon can also enhance the performance of these species in energy storage devices.^[^ ^31^ ^]^ Numerous examples in the literature,^[^ ^27^ ^]^ with no commercial application till now, have demonstrated the use of a variety of pillar ligands, which primarily include linear N‐donor organic ligands, and occasionally O‐donor ligands. However, they can also consist of purely inorganic linkers.^[^ ^32^ ^]^

The literature contains limited information on redox‐active pillared MOFs, suggesting a significant need for further investigation in this domain. Two examples of redox‐active pillared MOFs are reported in the literature: a) the two‐step formation of pillared MOFs using an electroactive trisphenylamine based compound, thereafter pillared by terephthalic acid linkers. This study was proved with a single crystal X‐ray diffraction analysis providing evidenced for a single‐crystal to single‐crystal (SC–SC) transformation^[^ ^33^ ^]^; b) a second reported example in the literature described the postsynthetic oxidation of a pillared MOF, where the metal center is involved in a redox reaction with I_2_.^[^ ^34^ ^]^ However, to the best of our knowledge, a one‐pot approach for the formation of microporous MOFs utilizing a redox‐active ligand, with its redox‐active center positioned on the pillar, has not been reported until now.

In this contribution, we employed the one‐pot strategy using solvothermal conditions in order to obtain robust MOFs, pillared by redox‐active ligands. Two series of coordination polymers were formed from two linear dicarboxylate‐based ligands (**H**_**2**_ **bpdc**, biphenyl‐4,4′‐dicarboxylic acid, and **H**_**2**_ **pda**, 1,4‐phenylenediacrylic acid) and one redox‐active linear N‐donor ligand, **L** (Figure 1), presenting the same metrics. These multifunctional series were characterized structurally, and adsorption and electrochemical properties were investigated for some of them.

## 2 Results and Discussion

**Scheme 1:**
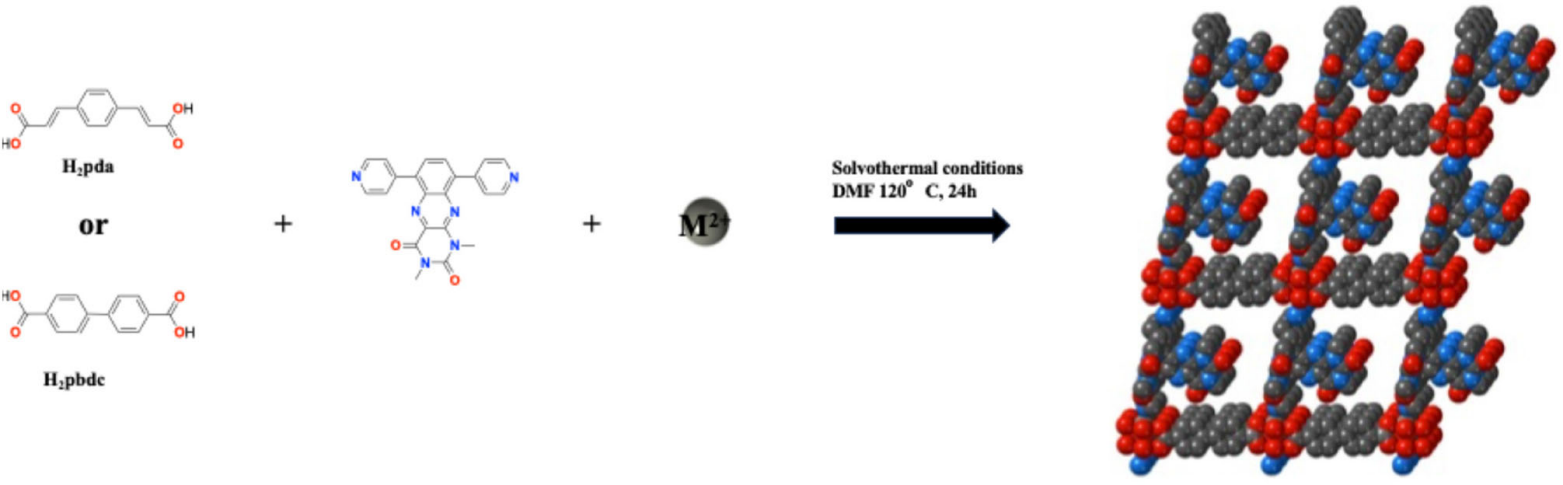
Synthetic pathway for the obtention of **1‐M** (M = Co, Ni, Cu, and Zn) and **2‐M** (M = Co or Cu).

Two separate alloxazine‐based redox‐active coordination polymer series were obtained by reacting a metal salt and **L** ^[^ ^23^ ^]^ with either **H**_**2**_ **pbdc** or **H**_**2**_ **pda,** which will be referred to as **1‐M** and **2‐M**, respectively (where M represents a particular metal from a specific metal salt), in solvothermal conditions as described in the experimental section, as shown in **Scheme** **1**. Each of them was characterized comprehensively, and the characteristics of each compound are individually described below.

### 2.1 Descriptions of the Structures

The combination of **L** with Zn^II^(NO_3_)_2_.*x*H_2_O and **H**_**2**_ **pbdc** in a 1:2:2 stoichiometric ratio, in DMF at 120 °C using solvothermal conditions, produced single crystals in the highest observed yield, for all metal salt. The single crystals were further investigated with single‐crystal X‐ray diffraction (SXRD). SXRD analysis revealed that the compound crystallizes in the space group *C*2/*c* (**Table** **1**) with the general formula [Zn_2_(pbdc)_2_ **L**]·2DMF (hereafter referred to as **1‐Zn**). The asymmetric unit is composed of two **pbdc** ^**2−**^ ligands, one **L** ligand, two free DMF molecules and two Zn^2+^ cations (Figure S1, Supporting Information). There is no electron exchange between **L** and Zn^2+^. Furthermore, one pyridyl ring of **L** is disordered. The XRD data are in accordance with the presence of neutral **L** and Zn^2+^ cations, meaning that in the MOF, there is no electron transfer between both moities.

**Table 1:** Crystallographic data for **1‐Zn** and **2‐Co**, measured at 120 K.

	**1‐Zn**	**2‐Co**
CCDC	**2426589**	**2394647**
Empirical formula	C_50_H_32_N_6_O_10_Zn_2_, 2(C_3_H_7_NO), solvent	C_46_H_32_Co_2_N_6_O_10_, solvent
Formula weight	1153.75	946.63
Crystal system, space group	Monoclinic *C*2/c	Triclinic, *P*‐1
Unit cell dimensions	*a* = 38.453(2) Å*b* = 21.9316(12) Å*c* = 21.0819(12) Å	*a* = 15.343(3) Å*b* = 15.420(2) Å*c* = 18.080(3) Å
	*α *= 90°*β *= 111.642(2)°*γ *= 90°	*α *= 96.526(5)°*β *= 92.575(5)°*γ *= 104.604(5)°
Volume	16,525.9(16) Å^3^	4100.6(11) Å^3^
*Z*, Calculated density	8, 0.927 Mg m^−^^3^	2, 0.767 Mg m^−^^3^
Absorption coefficient	0.626 mm^−1^	0.440 mm^−1^
F(000)	4752	968
Crystal size	0.200 × 0.200 × 0.140 mm	0.160 × 0.140 × 0.120 mm
Theta range for data collection	1.945 to 27.920 deg.	1.983 to 28.102 deg.
Limiting indices	−49 <= *h* <= 50,−28 <= *k* <= 28,−27 <= *l* <= 27	−20 <= *h* <= 20,−20 <= *k* <= 20,−23 <= *l* <= 20
Temperature	120(2) K	120(2) K
Wavelength	0.71073 A	0.71073 A
Reflections collected / unique	192,682/19,677 [R(int) = 0.0536]	111,585/19,099 [R(int) = 0.0978]
Completeness to theta =	99.5%	97.1%
Max. and min. transmission	0.7456 and 0.6892	0.7456 and 0.5416
Data / restraints / parameters	19,677/8/721	19,099/31/671
Goodness‐of‐fit on *F* ^2^	1.066	1.051
Final R indices [*I* > 2sigma(*I*)]	R1 = 0.0787, wR2 = 0.2295	R1 = 0.0940, wR2 = 0.2776
R indices (all data)	R1 = 0.1009, wR2 = 0.2517	R1 = 0.1265, wR2 = 0.3020
Largest diff. peak and hole	2.145 and −1.143 e.Å^−3^	1.426 and −1.314 e.Å^−3^

**Figure 2:**
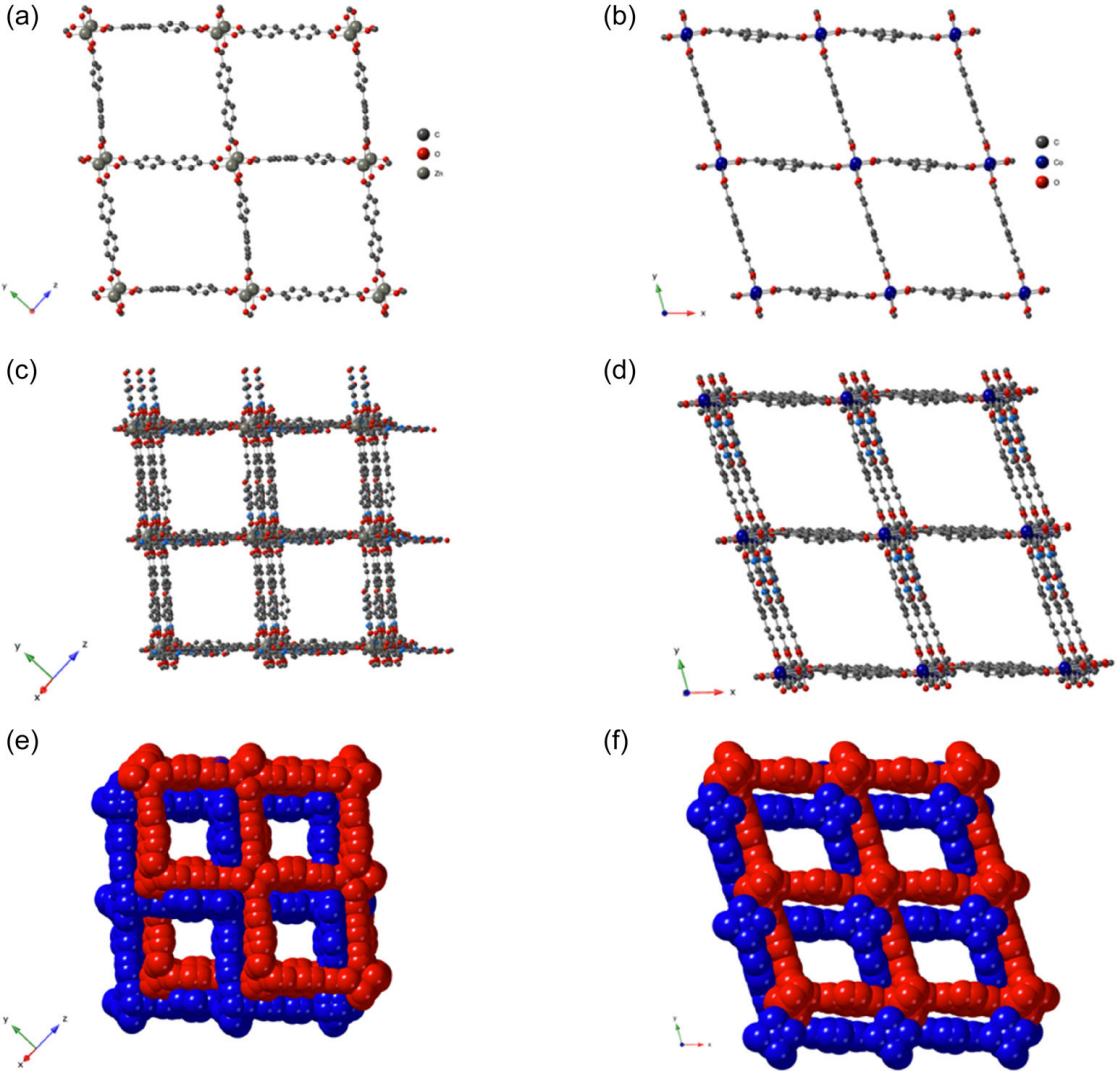
The X‐ray crystal structures of **1‐Zn**, a,c,e) representation in the [101] plane, and b,d,f) **2‐Co** in the x0y plane showing: (a,b) the 2D grid formed by the dicarboxylate ligands; (c,d) the connectivity of the 2D layers with the pillared ligands thus forming the 3D compounds; (e,f) the interpenetration of two 3D compounds, thus forming the resulting crystal structure. Disorder for **1‐Zn** and **2‐Co**, as well as the disordered **bpdc** ^**2−**^ and **pda** ^**2−**^ moieties in **2‐Co** are represented. H atoms were omitted for clarity.

The connectivity of this compound can be described as the formation of Zn_2_ paddlewheels (Zn–Zn distance of 2.9267(6) Å), (Table S1, Supporting Information). These Zn_2_ paddlewheels serve as slightly deformed square connecting units surrounded by four disordered **pbdc** ^**2−**^ ligands, ensuring the 1:1 metal: **pbdc** ^**2−**^ stoichiometric ratio. This results in the formation of a 2D lozenge (close to a deformed square grid) geometrical arrangement, with sides of lengths 15.199(6) and 15.222 (6) Å, and forming an angle of 87.5° (**Figure** **2**a). These 2D “grids” stack in layers, which are connected by the **L** ligands, thus forming a pillared 3D network with a metal:**pbdc**:**L** stoichiometric ratio of 2:2:1, as shown in Figure 2c. From the stacking of the 2D “grids,” channels are formed. The Zn^2+^ cations are in a deformed square pyramidal geometrical environment (Table S1 and Figure S2, Supporting Information).

In the crystal structure, two 3D networks are interpenetrating, as shown in Figure 2e, reducing the diameter of the channels to *ca* 9 Å. The two networks interact with each other through *π*‐*π* interactions involving pteridine (**L**) cores and pyridyl (**L**), as well as phenyl from the **pbdc** ^**2−**^ ligand as shown in Figure S3, Supporting Information.

The presence of diffuse solvent molecules inside the 3D networks, as frequently observed in MOFs, required us to use the squeeze command of Platon,^[^ ^35^ ^]^ and 248 residual electrons were found in the unit cell, corresponding to 6 molecules of DMF. This was in addition to the 2 crystallographically modeled DMF molecules, resulting in a total of 8 DMF molecules per unit cell. There are no interactions between the refined solvent molecules and the host network.

Analogous preparations were carried out, and single crystals of the Co analogue of **1‐Zn** were also obtained. However, only the cell parameters were refined, owing to poorly diffracting crystals (see Table S3, Supporting Information).

Using the same experimental conditions and incorporating **H**_**2**_ **pda** instead of **H**_**2**_ **bpdc**, and a Co salt, single crystals of **2‐Co** were also obtained. SXRD analysis revealed a compound presenting the molecular formula: **2‐Co** to be [Co_2_(pda)_2_ **L]**·DMF, with the compound crystallizing in the space group, *P*‐1 (Table 1). Its asymmetric unit is composed of two **pda** ^**2−**^ ligands, one **L** ligand, and two Co^2+^ cations (Figure S1, Supporting Information). The connectivity in **2‐Co** is analogous to the one observed in **1‐Zn**: a paddlewheel with a Co–Co distance of 2.6333(8) Å and a square pyramidal shape for the Co geometrical environment (see Table S1 and Figure S2, Supporting Information) is observed. The **pda** ^**2−**^ ligands similarly form lozenges with metal‐ligand‐metal distances measuring 15.0343(3) and 15.420 Å, and the smallest angle measuring 75.4° (Figure 2b,d and S2, Supporting Information). As with **1‐Zn**, both interpenetrated networks interact with each other by *π*–*π* interactions between two pteridine (**L**) moieties and the phenyl ring of the **pbdc** ^**2−**^ ligand (Figure S3, Supporting Information). The crystal structure contains channels with a diameter of ≈9 Å. (Table S1, Supporting Information).

Once again, the squeeze command was applied, and 280 residual electrons were found, corresponding to 7 molecules of DMF in the unit cell.

### 2.2 Powder X‐Ray Diffraction

**Figure 3:**
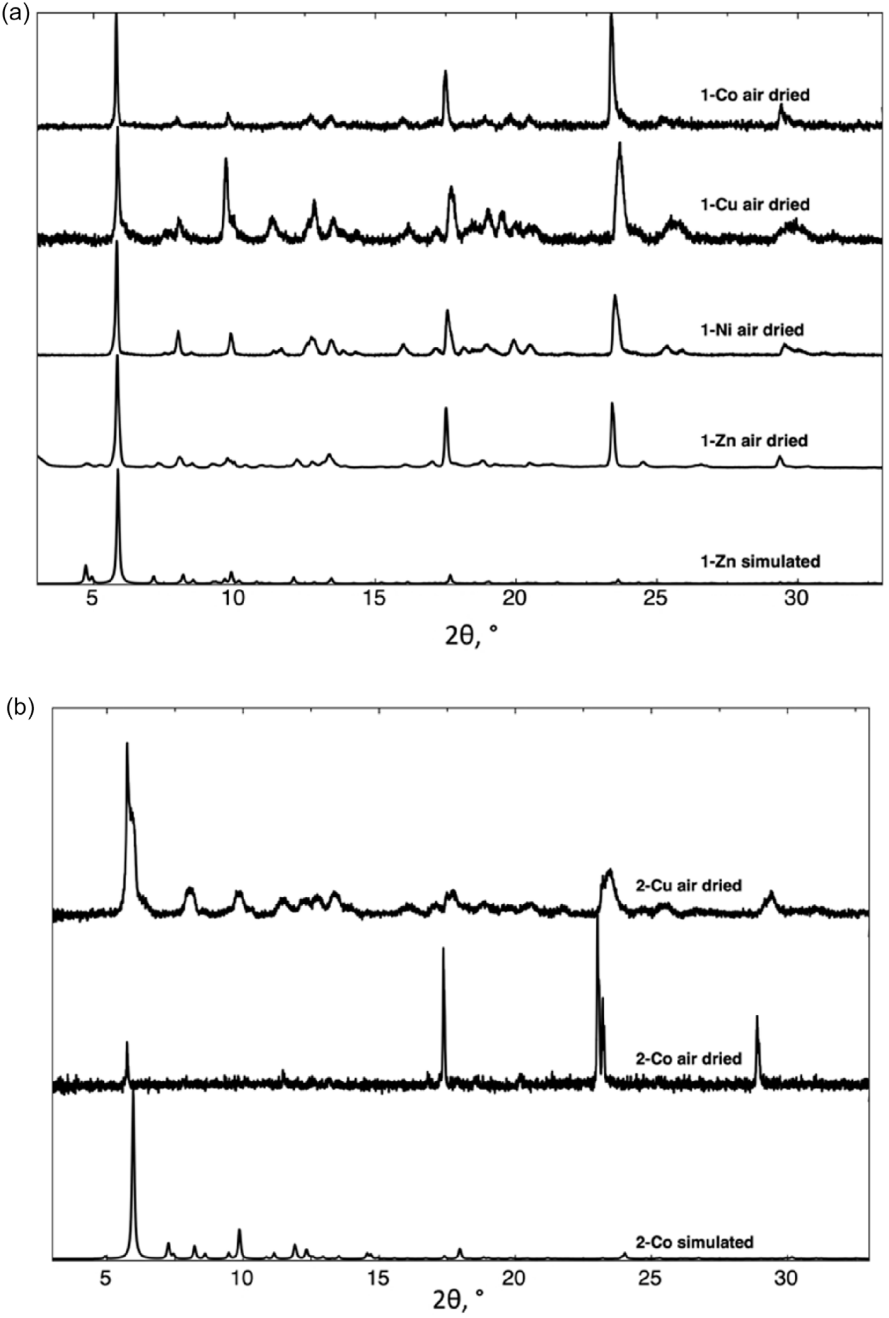
PXRD diffractograms for the air‐dried compounds a) **1‐M** series (M = Co, Ni, Cu, or Zn), compared to that calculated from the SXRD data for **1‐Zn**, and b) **2‐M** series (M = Co or Cu), compared to that calculated from the SXRD data observed for **2‐Co**. For all compounds, discrepancies in intensities between the observed and simulated diffractograms are due to preferential orientations of the microcrystalline powders. In addition, simulations have been performed on squeezed structures.

The microcrystalline samples of **1‐Zn** and **2‐Co** were also investigated using powder X‐ray diffraction (PXRD). After being exposed to the air, **1‐Zn** and **2‐Co** display similar phases as the simulated one, obtained from SCXRD data, as seen in **Figure** **3**a,b. The discrepancies between the 2*θ* peak positions of the simulated (from SCXRD data) versus air‐dried diffractograms could be as a result of different solvent quantities in the crystal structure, as well as the preferential orientation of the crystallites. The microcrystalline compounds also present similar phases to those of the single crystals of **1‐Zn** or **2‐Co**.

Attempts at obtaining isostructural compounds to **1‐Zn** and **2‐Co** were also performed using other metal salts (see Experimental Section). Additional isostructural coordination polymers were obtained as microcrystalline powders, as shown in Figure 3a (**1‐M**, M = Ni, Co and Cu) and Figure 3b (**2‐M**, M = Cu), and their PXRD diffractograms were compared with those observed for the powdered samples of **1‐Zn** (or **2‐Zn**). It shows that **1‐M** (M = Co, Ni, Cu, or Zn) and **2‐M** (M = Co or Cu) all contain similar phases, and thus the compounds in each series are isostructural. For **1‐Cu** and **2‐Cu**, the phases appear less crystalline.

### 2.3 Thermal Stability

Furthermore, the thermal stability of the series of compounds was evaluated using TGA. The measurements revealed high solvent losses; however, it was not possible to precisely determine the exact number of solvent molecules for each sample (i.e., the adsorbed H_2_O and DMF) due to the overlap in the loss of water and DMF in each of the TGA thermograms. Thereafter, the decomposition event occurred at around 400 °C for **1‐M** (M = Co, Ni and Zn), 400 °C for **2‐Co**, 320 °C for **1‐Cu,** and 280 °C for **2‐Cu** (refer to TGA thermograms, Figure S4, Supporting Information). For **1‐Zn** and **2‐Zn**) a relatively high decomposition temperature was observed.

### 2.4 Gas Sorption Properties and Behavior of the Network

After careful activation (see experimental section), the N_2_ adsorption measurements were performed on **1‐M** (M = Co, Ni, Cu and Zn) and **2‐M** (M = Co or Cu). The compounds indicate type I sorption profiles for N_2_ (see Figure S5, Supporting Information), and using the BJH model,^[^ ^36^ ^]^ the highest surface area of 645.9 m^2^.g^−1^ was determined for **1‐Zn**. The surface areas for all the isostructural compounds are provided in Table S4, Supporting Information, and they display strong differences, probably due to different behaviors of the compounds when activated.

**Figure 4:**
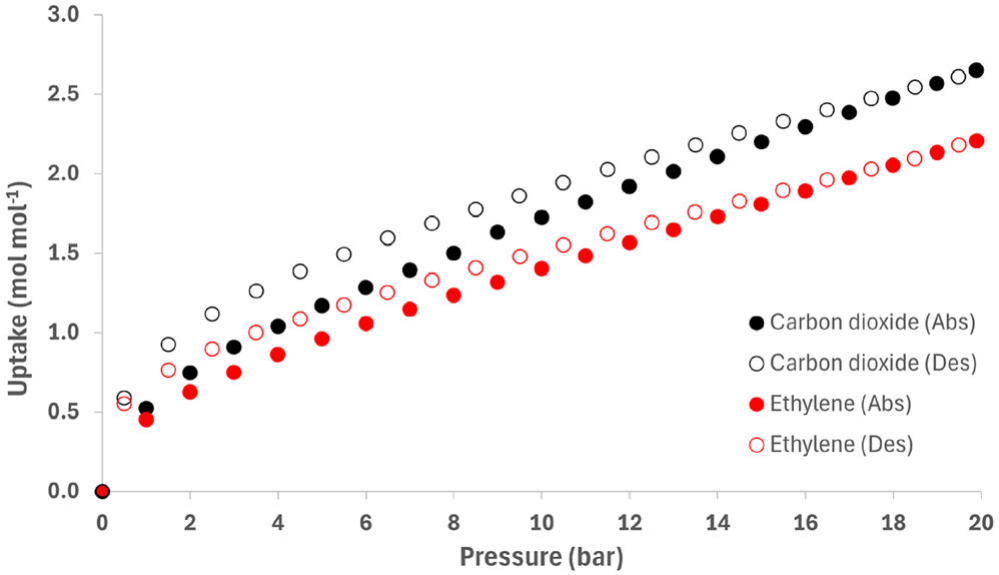
Isotherms of CO_2_ and C_2_H_4_ adsorption for **1‐Zn**, measured at T = 283 K.

In addition, high pressure gas sorption isotherms were recorded for **1‐Zn** with CO_2_ and C_2_H_4_ up to 20 bar (**Figure** **4**). Both experiments displayed type I sorption profiles over this pressure range, with minimal hysteresis during desorption. The maximum uptakes of CO_2_ and C_2_H_4_ at 20 bar amounted to 2.6 and 2.2 mol mol^−1^, respectively. Furthermore, it is evident from these results that the maximum uptake capacity for both gases has not been reached at 20 bar, as their respective sorption isotherm profiles have not yet plateaued. Lastly, the crystallinity of activated **1‐Zn** was retained after the sorption experiment, as indicated by comparison of the PXRD diffractograms of **1‐Zn** before and after the sorption experiments (Figure S6, Supporting Information), in addition, the positions of the peaks indicate that the scaffold of the MOF is preserved.

### 2.5 Electrochemical Behavior in the Solid State of 1‐M

The electrochemical properties of **1‐M** (M = Co, Cu, or Zn) were investigated. The electrochemical properties of **L** in solution, which have already been reported,^[^ ^23^ ^]^ showed that on a glassy carbon electrode in DMF solutions with TBABF_4_, **L** exhibits a first reduction wave at *E*_Red1 _= −0.80 V (*vs* Ag/AgCl), that has been attributed (CV coupled by EPR) to the formation of the radical anionic species, which is well‐known for alloxazine derivatives (see Figure S7, Supporting Information), where the charges are compensated either by protons (in the present case), or by monovalent metal ion (in a Metal‐ion cell, for example).

**Figure 5:**
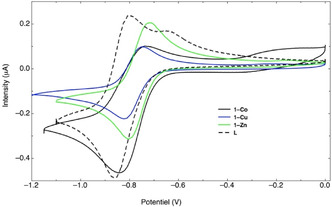
Cyclic voltammograms of the first reduction for (i) solution of **L** 1 mM in DMF with 0.1 M TBABF_4_: working electrode = glassy carbon, reference electrode = Ag/AgCl_sat_, counter electrode = Pt. Scan rate: 40 mV s^−1^ (blue color), and (ii) of solid‐state **1‐M** (M = Co, Cu or Zn) deposited on the glassy carbon electrode (black color).

**1‐M** (M = Co, Cu or Zn) has been investigated in the solid state by drop‐casting crystals of **1‐M** on a glassy carbon electrode immersed in DMF (refer to Experimental Section and **Figure** **5**).

The compounds present a reversible reduction peak located between *E* = −0.8 and −0.85 V (versus Ag/AgCl). In addition, all the compounds present, as observed for **L**, an additional irreversible reduction peak at lower potential. For the compounds investigated in the solid‐state, (**1‐M** (M = Co, Cu, or Zn), the *E*_Red1_ values for the reversible reduction process, which slightly varies between the 3 compounds, suggests that it is attributed to the reduction of the ligand, and the formation of a radical anionic species, as shown in Figure S7, Supporting Information. Due to their very different potential, it cannot be attributed to the reduction of the metal. Between the 3 compounds, there is a slight anodic shift, depending on the nature of the metal in **1‐M**.

For the first reduction, the broadening of the peaks, compared to that observed in solution for **L**, is probably due to low electrochemical processes while crystals are deposited at the surface of the working electrode.

Unfortunately, due to a small amount (films) of reduced compound in the solid state, further characterization (PXRD, gas sorption or TGA) of the reduced or cyclated MOF species could not be performed.

## 3 Conclusion

The first one‐pot pillared MOFs obtained using a redox‐active ligand are reported in this work. Two series of isomorphous compounds, presenting the same connectivity, are reported here: **1‐M** (M = Co, Ni, Cu or Zn) and **2‐M** (M = Co and Cu). The formation of **1‐M** is based on the use of **H**_**2**_ **bpdc**, biphenyl‐4,4′‐dicarboxylic acid, and for **2‐M**, the use of **H**_**2**_ **pda**, 1,4‐phenylenediacrylic acid. The resulting MOFs are robust compounds, pillared by N‐donor redox‐active species, based on the redox‐active alloxazine moiety. The fact that we obtain series of compounds accounts for the validity of the “pillars” approach. Both series of compounds represent the second example of coordination polymers involving alloxazine derivatives, and the first pillared MOFs using redox‐active pillars.

The compounds are thoroughly characterized from a structural point of view, revealing the formation of 2D deformed square or lozenge‐like networks, pillared by the linear alloxazine **L** ligands. This resulted in MOFs forming channels of *ca* 9 Å, and presenting the interpenetration of two 3D systems. The solvents present inside the cavity could not be all refined, due to being very disordered.

When desolvated, the compounds were subjected to N_2_ adsorption, revealing rather low surface areas. However, when exposed to air (for **1‐M** [M = Co, Ni, Cu, or Zn] and **2‐M** [M = Co or Cu]), the crystallinity is preserved, evidencing their robustness. In addition, the sorption isotherms of **1‐Zn** with CO_2_ and C_2_H_4_ indicate that there is potential for larger uptake at higher pressures, as the capacity of **1‐Zn** for both gases has not been reached. In fact, both absorption isotherms do not display any indication of reaching a plateau, but rather display a steady continuous uptake of gas at higher pressures. These results warrant further investigation in this area to determine the maximum capacity of this material.

In the solid‐state, the **1‐M** (M = Co, Cu or Zn) compounds display an electrochemical behavior suggesting that the redox‐process is centered on the ligand.

These observations, in relation with fundamental research, allow us to conclude that, using a three‐components approach, multifunctional redox‐active MOFs presenting channels and robust structures are formed and their electrochemical behavior was provided by the presence of the electroactive alloxazine moieties.

The compounds could have the potential to be implemented aspart of the electrode material in energy storage devices, like Li‐ion batteries and their corresponding electrochemical properties are currently under investigation.

## 4 Experimental Section

4.1

4.1.1

##### Synthetic Procedures

**L** was obtained following an already reported procedure.^[^ ^23^ ^]^ All the compounds are formed using classical solvothermal conditions. For all the compounds, due to a large amount of solvent present inside the cavities that is released when the crystals are exposed to air due to dynamical process, the elemental analysis data are not reproducible and are not shown here.

##### **1**‐**Z** **n**

**L** (20 mg, 0.05 mmol, 1 eq.), Zn(II)nitrate hexahydrate (29 mg, 0.1 mmol, 2 eq.), and **H**_**2**_ **pbdc** (22 mg, 0.1 mmol, 2 eq.) in 6 mL DMF were placed inside a sealed glass vessel. The vessel was heated at 120 °C for 24 h, and after 2 days of cooling, dark green plate‐like single‐crystals formed in 54% yield, which were suitable for SXRD analysis.

IR (KBr, cm^−1^) *υ*: 1661(s), 1609 (m), 1387 (s), 1092 (w), 823 (w), 772(s), 686 (m), 661 (m), 464 (s) (see Figure S7, Supporting Information).

##### **1**‐**Co**

The same procedure was followed by replacing Zn(II)nitrate hexahydrate by Co(II)nitrate hexahydrate (30 mg, 0.1 mmol, 2 eq.). Yield 72%.

IR (KBr, cm^−1^) *υ*: 1660 (s), 1564 (w), 1384 (s), 1357 (m), 1252 (w), 1097 (m), 781 (m), 658(m), 487(s) (see Figure S8, Supporting Information).

##### **1**‐**Cu**

The same procedure was followed by replacing Zn(II)nitrate hexahydrate by Cu(II)nitrate hexahydrate (30 mg, 0.1 mmol, 2 eq.). Yield 72%.

IR (KBr, cm^−1^) *υ*: 1660 (s), 1563 (w), 1387 (s), 1357 (m), 1250 (w), 1098 (m), 779 (m), 659(m), 488(s) (see Figure S8, Supporting Information).

##### **1**‐**Ni**

The same procedure was followed by replacing Zn(II)nitrate hexahydrate by Ni(II)nitrate hexahydrate (30 mg, 0.1 mmol, 2 eq.). Yield 72%.

IR (KBr, cm^−1^) *υ*: 1660 (s), 1565 (w), 1383 (s), 1357 (m), 1251 (w), 1096 (m), 780 (m), 659(m), 487(s) (see Figure S8, Supporting Information).

##### **2**‐**Co**

**L** (20 mg, 0.05 mmol, 1 eq.), Co(II)nitrate hexahydrate (29 mg, 0.1 mmol, 2 eq.), and **H**_**2**_ **pda** (22 mg, 0.1 mmol, 2 eq.) in 6 mL DMF were placed inside a sealed glass vessel. The vessel was heated at 120 °C for 48 h and dark green plate‐like single‐crystals suitable for SXRD formed after 2 days. Yield 32%.

IR (KBr, cm^−1^) *υ*: 1655 (s), 1591 (m), 1493 (w), 1383 (s), 1253 (w), 1091 (m), 1061 (w), 979 (w), 816 (w), 713 (w), 655 (m) (see Figure S9, Supporting Information).

##### **2**‐**Cu**

The same procedure was followed by replacing Co(II)nitrate hexahydrate by Cu(II)nitrate trihydrate (24 mg, 0.1 mmol, 2 eq.). The vessel was heated at 120 °C for 2 h and a light green powder was formed after 4 days. Yield 53%.

IR (KBr, cm^−1^) *υ*: 1652 (s), 1590 (m), 1493 (w), 1384 (s), 1253 (w), 1093 (m), 1061 (w), 980 (w), 820 (w), 712 (w), 658 (m) (see Figure S9, Supporting Information).

##### Materials and Methods

FT‐IR spectra of the powdered microcrystalline samples were recorded on a Perkin Elmer Spectrum Two FTIR‐UATR in the wavenumber interval of 4000–400 cm^−1^.

##### Gas Sorption Measurements

Low‐pressure gas sorption measurements (N_2_) were obtained on a Micromeritics ASAP 2020 analyzer. Powdered microcrystalline samples were activated under dynamic vacuum at a temperature optimized for each material. High‐pressure sorption experiments were performed using a Hiden Isochema IGA‐001 gravimetric sorption analyzer, equipped with a Grant refrigerated circulating bath to maintain the temperature at 10 °C. Data were recorded up to 20 bar with an equilibration time‐out setting of 150 min. **1‐Zn** was activated at 160 °C for 8 h under dynamic vacuum.

##### TGA Measurements

TGA measurements were performed on powdered microcrystalline compounds on a Pyris 6 TGA Lab System (Perkin‐Elmer), using a N_2_ flow of 20 mL min^−1^ and a heat rate of 4 °C min^−1^.

##### Electrochemical Procedure

The electrochemical measurements were carried out at RT (20 °C) in DMF containing 0.1 M TBABF_4_ in a classical three‐electrode cell. The working electrode was a 3 mm glassy carbon disk, the counter electrode was a Pt wire, and an Ag(s)|AgCl(s) electrode was selected as a reference. The electrolyte was degassed by bubbling argon through the solution for at least 10 min, and the argon flow was kept over the solution during the measurements. The electrochemical cell was connected to a computerized multipurpose electrochemical device (BIOLOGIC potentiostat, model SP‐150). Cyclic voltammetry experiments were performed at a scan rate varying from 20 to 100 mV s^−1^. CVs that are presented are corrected for the ohmic drop.

The deposition of **1‐M** on the glassy carbon electrode was performed by suspending microcrystalline powder of **1‐M** (M = Co, Cu, or Zn) in an EtOH solution; then, the solution was dropcasted on the surface of the electrode and dried in air.

##### Crystallography: SXRD

Intensity data were collected for **1‐Zn** and **2‐Co**, using a 4‐circle Bruker PHOTON III diffractometer equipped with two micro‐sources IμS Mo and IμS Diamond Cu, along with an Oxford Cryosystem 800 for low temperature measurements. The cell parameters were determined using APEX3 software,^[^ ^37^ ^]^ and the structures were solved using the program SHELXT‐2014.^[^ ^38^ ^]^ The refinement and all further calculations were carried out using SHELXL‐2018.^[^ ^39^ ^]^ Hydrogen atoms were included in calculated positions and treated as riding atoms using SHELXL default parameters. The non‐H atoms were refined anisotropically, using weighted full‐matrix least‐squares on *F* ^2^. A semi‐empirical absorption correction was applied using SADABS in APEX3.

CCDC Numbers for **1‐Zn**: **2426589** and **2‐Co**: **2394648**.

##### Crystallography: PXRD

Diffractograms for the air‐dried microcrystalline samples were recorded at room temperature (293(2) K), on a Bruker D8 diffractometer using monochromatic Cu‐K*α* radiation. A scanning range between 2° and 40° with a scan step size of 2° min^−1^ was used, and the sample holder was rotated at 15 rpm.

## Supporting Information

For **1‐Zn** and **2‐Co** asymmetric units, environment of the metal centers, representation of the stacking in the unit cell, main distances. Refined cell parameters for **1‐Co**. For **1‐M** (M = Co, Ni, Cu, and Zn) and **2‐M** (M = Co or Cu), TGA traces, N_2_ adsorption and IR spectra in the solid state. For **1‐Zn**, PXRD after activation and after the sorption measurements. The three different electronic states for the alloxazine moiety.

## Conflict of Interest

The authors declare no conflict of interest.

## Supporting information

Supplementary Material

## Data Availability

The data that support the findings of this study are available from the corresponding author upon reasonable request.
