# Supplementary material for: Series of Microporous Redox‐Active Pillared Metal–Organic Frameworks Based On Alloxazine Ligands
Source: ChemistryOpen. 2025 Sep 9;14(12):e202500461. doi: 10.1002/open.202500461 (PMC12680567; doi:10.1002/open.202500461)
Supplement: Supplementary file 1 — Supplementary Material [file OPEN-14-e202500461-s001.pdf]

**Supporting Information**

# Series of microporous redox-active pillared MOFs based on alloxazine ligands

Jaison Casas,[a] Alexios I. Vicatos,[b] Leonard J. Barbour,[b] Nathalie Kyritsakas,[c] Abdelaziz Jouaiti,[a] Sylvie Ferlay*[a]

[a] Dr. Jaison Casas, Dr. Abdelaziz Jouaiti, Prof. Sylvie Ferlay
Université de Strasbourg, CNRS, CMC UMR 7140, F-67000 Strasbourg, France
E-mail: ferlay@unistra.fr

[b] Dr. Alexios I. Vicatos, Prof. Leonard J. Barbour
Department of Chemistry and Polymer Science, Stellenbosch University, Matieland 7602, South Africa

[c] Nathalie Kyritsakas
Service de radiocristallographie de la Fédération de Chimie Le Bel – UAR 2042, Université de Strasbourg and CNRS F-67000 Strasbourg, France



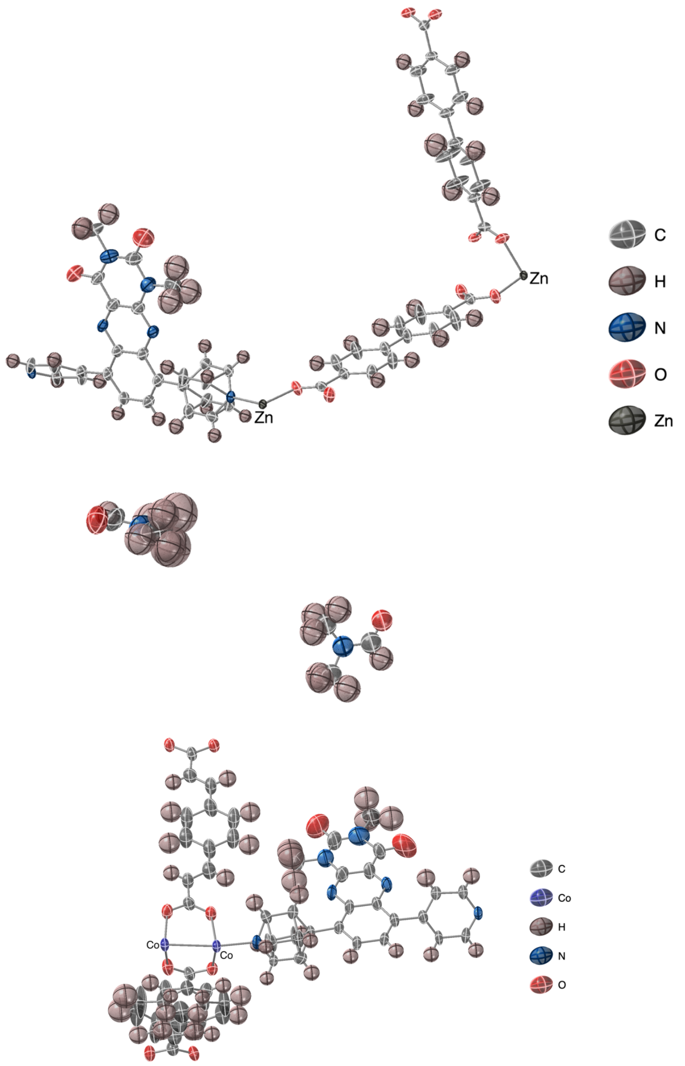


**Figures S1**: The asymmetric units for **1-Zn** (top) and **2-Co** (bottom). Thermal ellipsoids are at 50% probability.

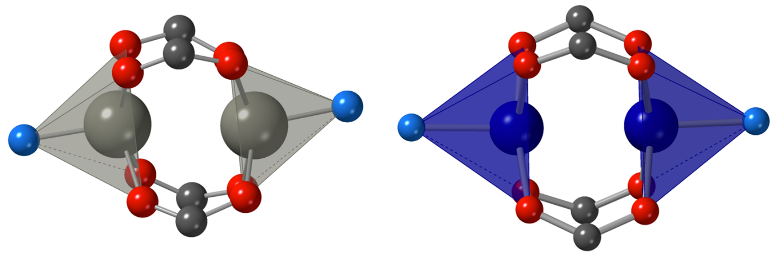

**a** **b**

**Figures S2**: Portion of the X-ray structure of **1-Zn** (a) in the x0y plane and **2-Co** (b) showing the environment around the metal centres.

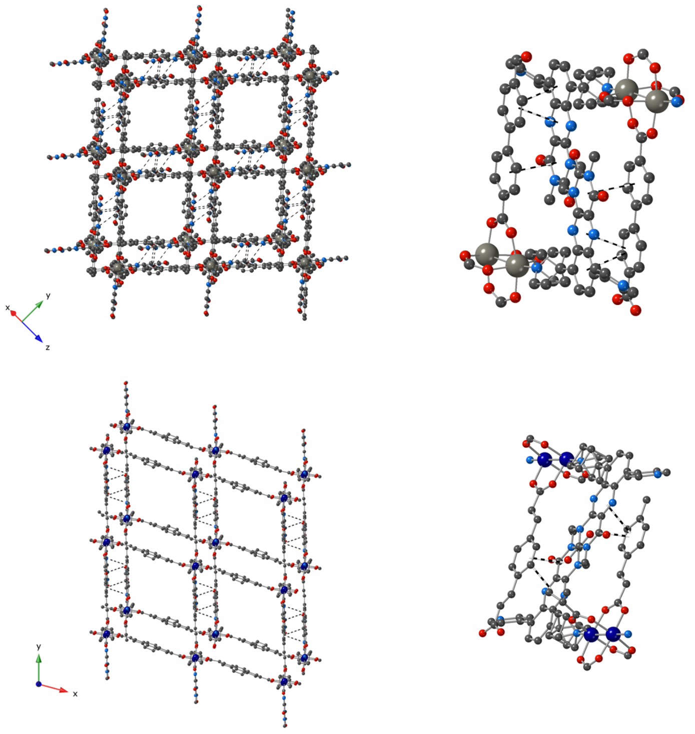

**Figures S3**: The detailed π- π interactions (represented as dashed lines) in **1-Zn** (top) and **2-Co** (bottom).



| | **1-Zn** | **2-Co** |
|---|---|---|
| M–O | 2.014(3)<br>2.031(3)<br>2.038(3)<br>2.043(3)<br>2.044(3)<br>2.045(3)<br>2.048(3) | 2.011(3)<br>2.014(3)<br>2.016(3)<br>2.017(3)<br>2.021(3)<br>2.024(3)<br>2.031(3)<br>2.036(3) |
| M–N | 2.026(3)<br>2.031(3) | 2.048(4)<br>2.058(4) |
| M-M | 2.9267(6) | 2.6333(8) |

**Table S2**. Selected bonds lengths (Å) around the M(II) ions for **1-Zn** and **2-Co**.

| | **1-Co** |
|---|---|
| Empirical formula | $C_{50}H_{32}N_6O_{10}Co_2$, 2($C_3H_7NO$), solvent |
| Formula weight | 1140.87 |
| Crystal system, space group | Monoclinic *C* 2/c |
| Unit cell dimensions | a = 37.147(4) Å<br>b = 22.158(2) Å<br>c = 20.9767(19) Å |
| | α = 90 deg.<br>β = 109.388(3) deg.<br>γ = 90deg. |
| Volume | 16287(3) Å$^3$ |

**Table S3**. Cell parameters determined for **1-Co**.

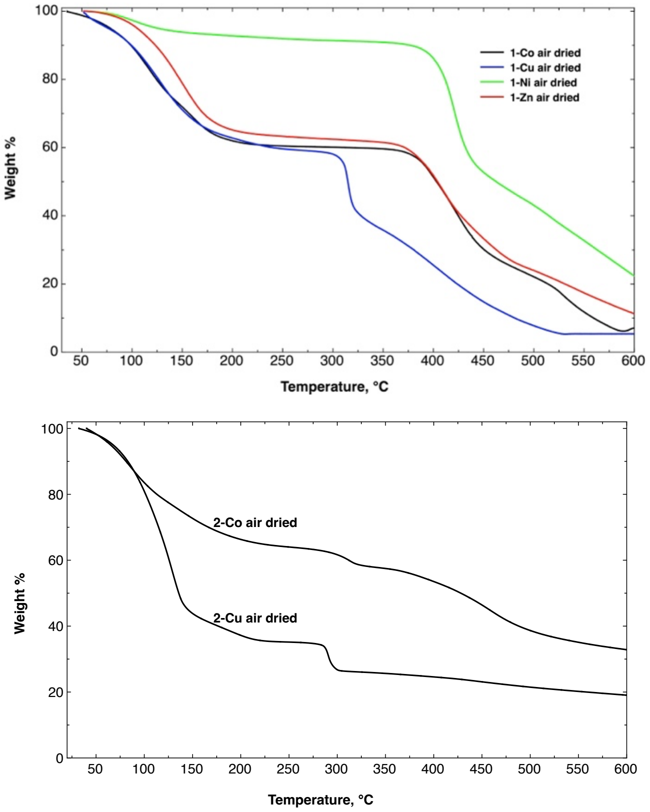


**Figure S4.** For **1-M** (M = Co, Cu, Ni and Zn) and **2-M** (M = Co or Cu), TGA thermograms between T = 30 °C and 500 °C.



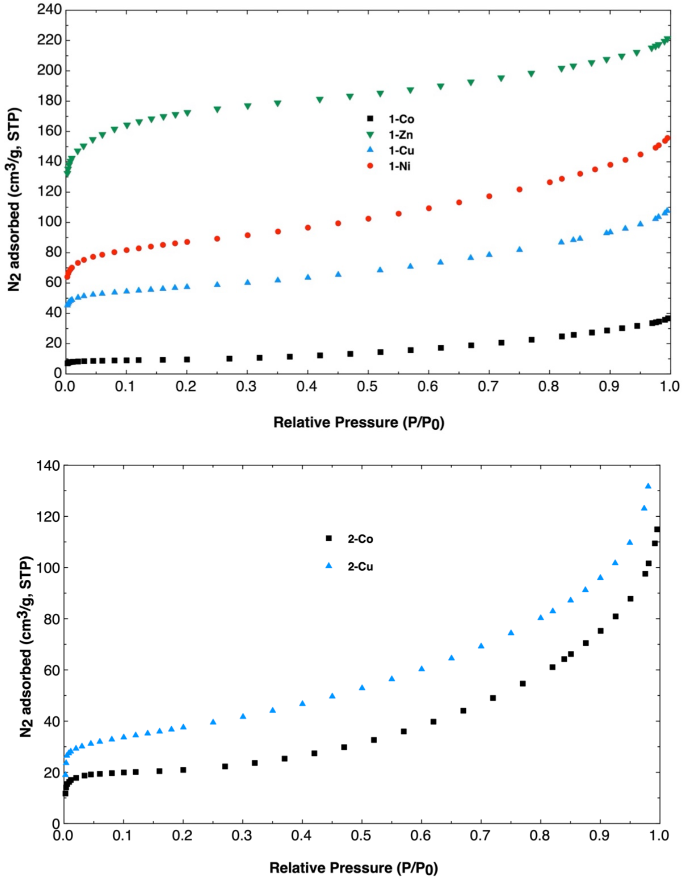


**Figure S5.** For **1-M** (M = Co, Cu, Ni or Zn) and **2-M** (M = Co or Zn), adsorption isotherms $N_2$ at T = 77K.

| | **1-Co** | **1-Zn** | **1-Cu** | **1-Ni** | **2-Co** | **2-Cu** |
|---|---|---|---|---|---|---|
| **SA ($m^2.g^{-1}$)** | 35.7 | 645.9 | 213.4 | 320.7 | 6.4 | 131.2 |

**Table S4**. Estimated surface areas for **1-M** (M = Co, Cu, Ni or Zn) and **2-M** (M = Co or Zn)

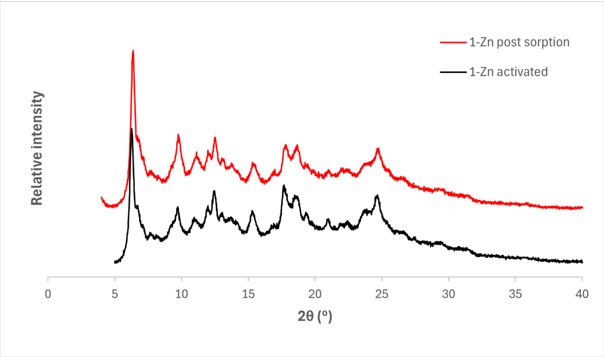


**Figure S6**: A comparison of the PXRD diffractograms of **1-Zn** activated before the sorption experiments, and **1-Zn** after the sorption experiments.

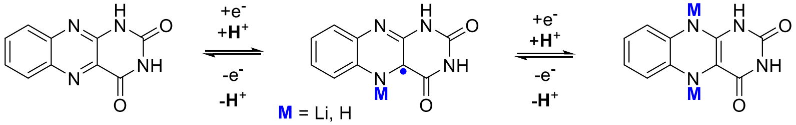


**Figure S7**: The three different electronic states of the alloxazine moiety and the associated electron/proton(metal) transferts.



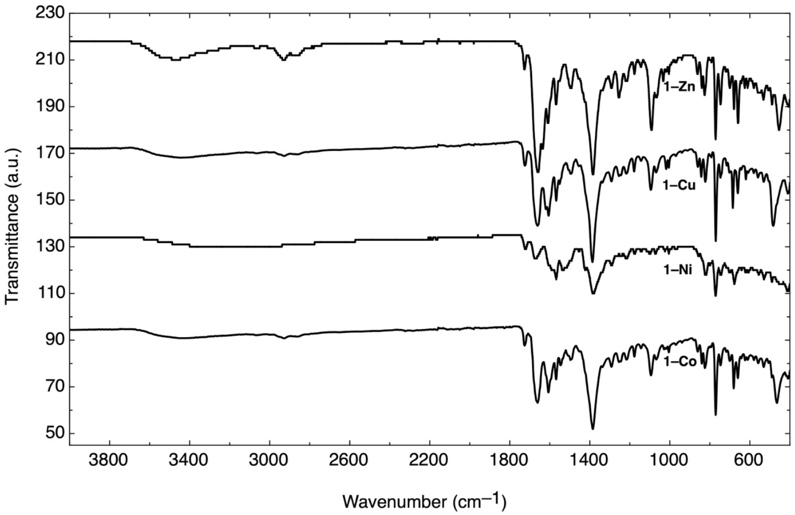


**Figure S8.** For **1-M** (M = Co, Cu, Ni and Zn), IR spectrum in the solid-state (ATR).

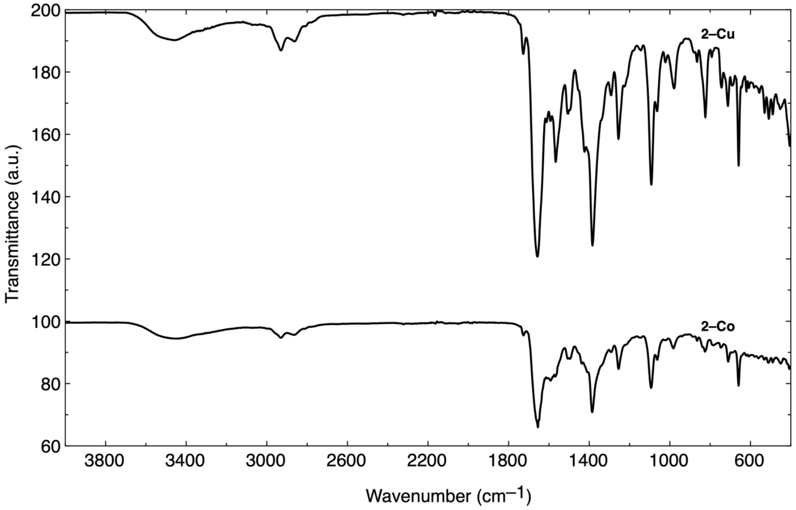


**Figure S9.** For **2-M** (M = Co or Cu), IR spectrum in the solid-state (ATR).
